# Lower school performance in late chronotypes: underlying factors and mechanisms

**DOI:** 10.1038/s41598-017-04076-y

**Published:** 2017-06-29

**Authors:** Giulia Zerbini, Vincent van der Vinne, Lana K. M. Otto, Thomas Kantermann, Wim P. Krijnen, Till Roenneberg, Martha Merrow

**Affiliations:** 1 University of Groningen, Groningen Institute for Evolutionary Life Sciences, Department of Neurobiology, Groningen, 9747AG NL The Netherlands; 2 University of Massachusetts Medical School, Department of Neurobiology, Worcester, MA 01655 USA; 3 Ludwig-Maximilians-University, Institute for Occupational, Social and Environmental Medicine, Munich, 80336 DE Germany; 4 University of Groningen, Faculty of Science and Engineering, Groningen, 9747AG NL The Netherlands; 5 Ludwig-Maximilians-University, Institute of Medical Psychology, Munich, 80336 DE Germany

## Abstract

Success at school determines future career opportunities. We described a time-of-day specific disparity in school performance between early and late chronotypes. Several studies showed that students with a late chronotype and short sleep duration obtain lower grades, suggesting that early school starting times handicap their performance. How chronotype, sleep duration, and time of day impact school performance is not clear. At a Dutch high school, we collected 40,890 grades obtained in a variety of school subjects over an entire school year. We found that the strength of the effect of chronotype on grades was similar to that of absenteeism, and that late chronotypes were more often absent. The difference in grades between the earliest 20% and the latest 20% of chronotypes corresponds to a drop from the 55^th^ to 43^rd^ percentile of grades. In academic subjects using mainly fluid cognition (scientific subjects), the correlation with grades and chronotype was significant while subjects relying on crystallised intelligence (humanistic/linguistic) showed no correlation with chronotype. Based on these and previous results, we can expand our earlier findings concerning exam times: students with a late chronotype are at a disadvantage in exams on scientific subjects, and when they are examined early in the day.

## Introduction

The gateway to success is education. What pupils learn and how they perform during primary and secondary education influences their future career opportunities^1^. Academic beliefs (e.g. perceived academic competence), motivation, and intelligence have been shown to play an important role in school performance^2, 3^. Other factors related to class and family environment such as teacher quality, socio-economic status, and parental involvement are also associated with school achievements^4–6^.

The role of sleep in relation to school performance has been extensively studied. Cognitive performance can be quantitatively impaired by sleep deprivation and high-school students usually carry more sleep debt than younger or older individuals^7–9^. Previously, we reported that students who are late chronotypes – those who sleep at the latest times of the day – perform worse on exams that are scheduled in the morning in comparison to those scheduled later in the day^10^. Importantly, early and late chronotypes in our study performed equally well in the afternoon. A number of reports have purported that either early or late chronotypes are more or less intelligent^11–13^. Based on the lack of agreement between these studies, their weak significance, and our previous findings, we assume that chronotype is not associated with intelligence. Chronotype can be assessed via the Munich ChronoType Questionnaire (MCTQ^14^) as the midpoint of sleep on work-free days (MSF). This value is further corrected for sleep debt accumulated on school/work days (MSF_sc_). Chronotype is predominantly controlled by the circadian clock and external timing signals (zeitgebers)^14^. Humans entrain (synchronise) with different phases to the external light-dark cycle, giving rise to a distribution of chronotypes, ranging from early (larks) to late (owls) types^15^. Chronotype varies with age and is latest during adolescence^16, 17^. Despite the late chronotype in adolescents, several schools start early in the morning (8:30 h on average in the Netherlands), leading to chronic sleep deprivation in most high-school students^18, 19^.

Late chronotype has been correlated with shorter sleep duration on school/work days^20^, and late types as well as short sleepers have been shown to obtain lower grades on average^10, 21^.

The influence of chronotype, sleep duration, and time of day on school performance has received some attention in previous studies. One possibility is that late chronotypes are tested at an earlier internal time (internal time can be expressed as hours since MSF_sc_) before they reach their peak performance. This is supported by our previous finding that the chronotype-effect on grades is pronounced in the early morning, but insignificant in the early afternoon^10^. Highly controlled laboratory experiments have found that cognitive abilities relying mainly on so-called fluid intelligence (e.g. logic, reasoning, problem solving) are susceptible to time-of-day and chronotype-effects^22^. Early chronotypes tend to perform better in the morning while late chronotypes perform better in the evening^13, 23^. Crystallised intelligence (e.g. general knowledge, long-term memory vocabulary), on the contrary, was found to be immune to time-of-day and chronotype-effects^24, 25^.

Another possible explanation for lower school performance in late chronotypes is that chronic sleep deprivation impairs cognitive abilities. Sleep deprivation can affect functioning of the prefrontal cortex and cortical-thalamic circuits, which are involved in controlling high-order cognitive functions, such as logic and reasoning, abstract thinking, and problem solving (fluid intelligence)^26, 27^. Although sleep supports memory consolidation, access to long-term-acquired knowledge (crystallised intelligence) seems to be less impaired by sleep deprivation compared to fluid intelligence^28–30^.

Chronotype could also be associated with other factors (e.g. school attendance) involved in determining school achievements. Absenteeism was found to correlate negatively with worse grades^31^, but research on the relationship between chronotype and school attendance/absenteeism is lacking. Early school starting times challenge late chronotypes more than early chronotypes, which could lead to more tardiness (e.g. due to oversleep), and more days of sick leave in late chronotypes with negative consequences for their school grades.

The aim of the current study is to explore if chronotype, sleep duration on school nights, and school attendance alone and in combination can predict school performance. We analysed grades obtained in Dutch high-school students over an entire school year. When considering this specific set of predictors of school performance, we found that chronotype had a stronger impact on grades than sleep duration. This association was strongest for scientific subjects. Absenteeism was increased in late chronotypes and was associated with an overall decrease in grades, independent of school subject.

## Results

A total of 40,890 grades from individual examinations taken throughout the school year by students attending the first three years of secondary education (523 students; average number of grades per student: 78; age range: 11–17 years) were collected. Of these students, 426 (219 females and 207 males, mean age 13.06 ± 0.95 SD; age range 11–16 years) had filled in the MCTQ to assess their chronotype, social jetlag, and average sleep duration on school nights (Table 1 and Supplementary Figure S1). In the Dutch secondary school system, grades range from 1 (lowest) to 10 (highest), with 6 considered to be the threshold to pass an examination^32^. The conversion between Dutch grades and US grades is the following: 1–5 = F, 5.5 = D, 6 = C, 6.5 = B, 7 = B+, 7.5–8 = A, 8.5–10 = A+. Data on school attendance were retrieved from the school’s registration system. The number and percentage of students absent from class together with the total counts of late arrivals, dismissals from class, sick leaves and duration of sick leaves are reported in Table 2.

**Table 1:** Demographics of 219 female and 207 male high school students attending the first three school years (data from the Munich ChronoType Questionnaire).

Outcome measure	Average (±SD)	Range
Age (years)	13.06 (0.95)	11–16
Chronotype (MSF_sc_, h:min)	4:16 (65′)	23:37–8:23
Social Jetlag (h:min)	2:13 (59′)	0–5:56
Sleep onset on school days (h:min)	22:37 (59′)	20:35–4:00
Sleep end on school days (h:min)	6:38 (25′)	4:30–8:15
Sleep duration on school days (h:min)	8:01 (63′)	3:15–10:40
Sleep onset on school-free days (h:min)	0:05 (81′)	21:10–5:15
Sleep end on school-free days (h:min)	9:32 (88′)	2:00–14:00
Sleep duration on school-free days (h:min)	9:26 (88′)	1:30–12:40

Data concerning chronotype, sleep onset and sleep end refer to external clock time and are reported in clock hours.

**Table 2:** Number and percentage (%) of students absent from class.

	All students		Students with MCTQ	
Outcome measure	N students (%)	Count	N students (%)	Count
Late arrivals	249 (47%)	589	197 (46%)	421
Dismissals from class	153 (29%)	353	120 (28%)	272
Sick leaves	395 (75%)	1,346	316 (74%)	1,025
Sick leave duration (d)	395 (75%)	2,259	316 (74%)	1,627

Number and percentage of students are reported separately for the entire student population (523), and for the students who filled in the MCTQ (426). The total counts of late arrivals, dismissals from class, sick leaves, and sick leave duration (days) are also reported.

**Figure 1:**
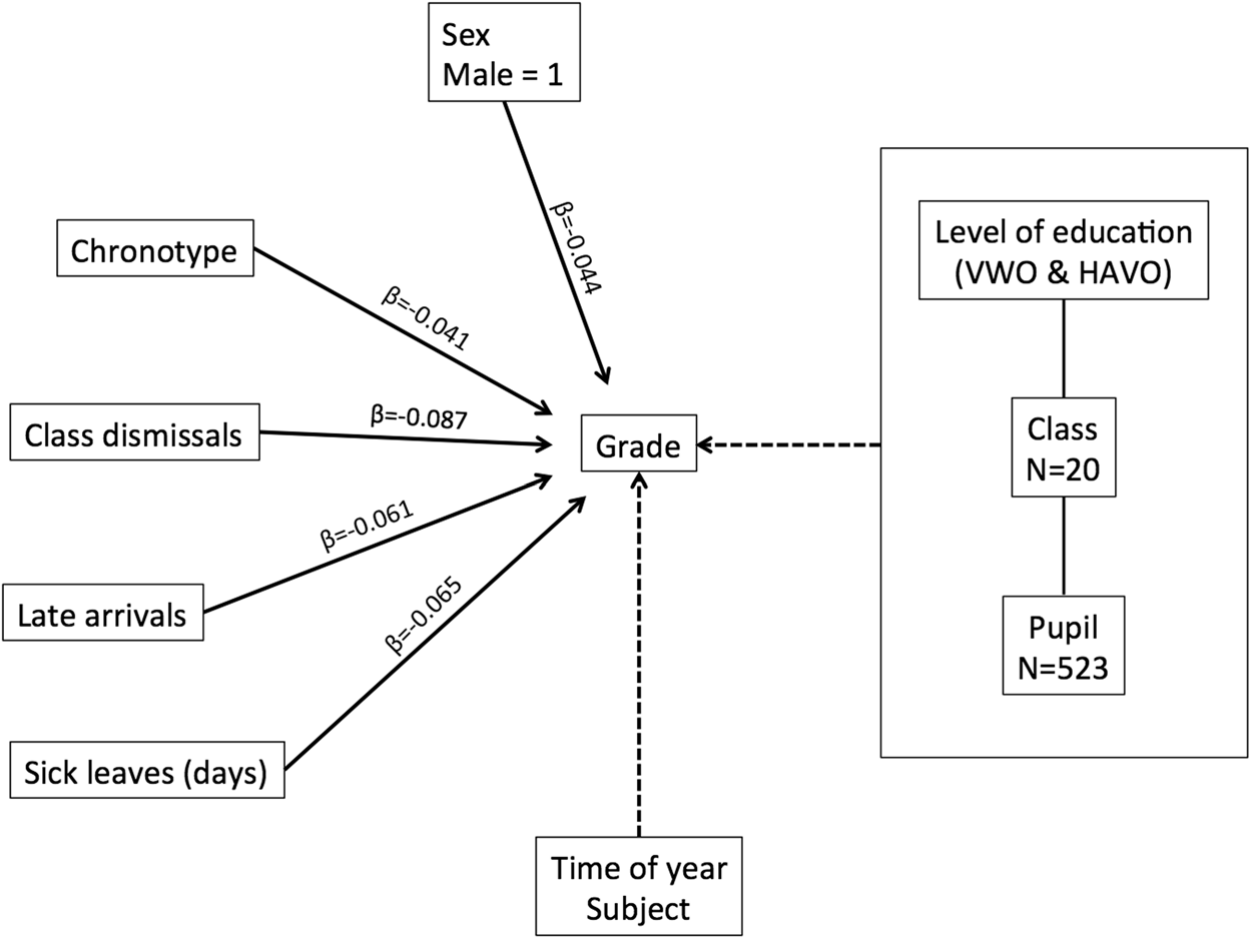
Multilevel model selected as the most parsimonious fit (according to the AIC) to explain the influence of the independent variables on school grades (dependent variable). The explanatory (independent) variables were: sex, chronotype (MSF_sc_), late arrivals during the first hour, class dismissals, and sick leaves (duration). The standardised beta coefficients (β) were negative for each variable and are reported on the solid connecting lines between independent and dependent variables. The interpretation of a negative beta coefficient is the following: for every 1-standard deviation increase in the explanatory variable, the standard deviation of the dependent variable will decrease by the beta coefficient value. For the variable ‘sex’, males were compared with females, meaning that a negative beta coefficient reflected a decrease in grades for males. Time of year and school subject were evaluated in the model as covariates (dashed connecting lines).

The influence of the explanatory variables (demographic, sleep-related, and school attendance variables) on school grades was assessed with a multilevel approach. Our analysis of model selection based on the AIC indicated that model 1 and model 4 (AIC model 1: 118909.8; AIC model 4: 118909) were the most parsimonious models to explain the variation in school grades. Both models had chronotype, sex, late arrivals during the first hour, dismissals from class, and sick leaves (duration) as predictors, and model 4 had age as additional predictor. Since age was found not to be significantly associated with grades, we report here the results obtained with model 1, following the principle of parsimony^33^ (Fig. 1; see Supplementary Table S2 for a detailed description of the 9 models, and the comparison of AIC values between models). We found that chronotype was negatively correlated with grades, with later chronotypes obtaining lower grades compared to earlier chronotypes (b = −0.060, t (407) = −2.313, p = 0.0212). A one-hour later chronotype correlated with an overall decrease in grades with a factor of 0.06 (on a scale from 1 to 10). Sex also had a significant effect on grades (b = −0.138, t (407) = −2.542, p = 0.0114), with males obtaining lower grades (on average 0.14 lower) compared to females. School attendance was found to be associated with grades, with increased absenteeism negatively impacting grades (late arrivals: b = −0.062, t (407) = −3.283, p = 0.0011; dismissals from class: b = −0.090, t (407) = −4.608, p < 0.0001; sick leave duration: b = −0.019, t (407) = −3.875, p = 0.0001). Our model predicted an overall decrease in grades of 0.09 for a student dismissed from class one time, a decrease of 0.06 for a student arriving late one time, and a decrease of 0.02 for a student being sick one day in the course of an entire school year. To compare the strength of the effects of this set of predictors, we calculated the standardised beta (β) coefficients (Fig. 1). The effect on grades was stronger for dismissals from class (β = −0.087), followed by sick leave duration (β = −0.065), late arrivals (β = −0.061), sex (β = −0.044), and chronotype (β = −0.042).

Short sleep duration was found to be significantly associated with lower grades only in model 9 (b = 0.083, t (423) = 2.943, p = 0.0034). This model did not include the school attendance variables as predictors and had the highest AIC value (worse fit) among all models considered. Similarly, increased levels of social jetlag were significantly associated with lower grades only in model 8 (b = −0.094, t (409) = −3.188, p = 0.0015), which did not include the school attendance variables as predictors.

The mediation analysis showed that the direct effect of chronotype on grades was significant (late arrivals: b = −0.085, p = 0.01; dismissals from class: b = −0.071, p = 0.01; sick leave (duration): b = −0.087, p < 0.01; sleep duration on school days: b = −0.081, p < 0.01), while the indirect effect of chronotype mediated by late arrivals, dismissals from class, sick leave (duration), and sleep duration on school days was not (late arrivals: b = −0.005, p = 0.32; dismissals from class: b = −0.006, p = 0.15; sick leave (duration): b = −0.005, p = 0.12; sleep duration on school days: b = −0.015, p = 0.09).

**Figure 2:**
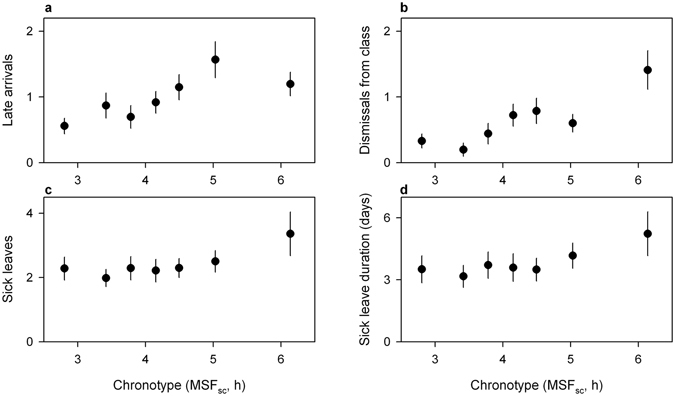
Influence of chronotype (MSF_sc_) on school attendance. Data points represent mean number of late arrivals (**a**), dismissals from class (**b**), sick leaves (**c**) and days of sick leave (**d**) with standard error of the mean (SEM). The averages were calculated over the entire school year. The students were divided into 7 equal-sized groups based on chronotype (lower numbers correspond to earlier chronotypes and higher numbers to later chronotypes, respectively). Late chronotypes were significantly more likely to arrive late, be dismissed from class, become sick, and miss more days due to sickness.

In addition to the influence on grades, we found that chronotype was related to school attendance (Fig. 2a–d). Chronotype influenced the likelihood of arriving late to the first lesson of the day (b = −0.695, z = −2.555, p = 0.0106; Fig. 2a). For instance, a student with a chronotype of 3 had two times larger odds of never being late compared to a student with a chronotype of 4 (MSF_sc_ of one hour later). Age influenced the frequency of late arrivals, with older students arriving late more often compared to younger students (b = 0.320, z = 4.700, p < 0.0001). Later chronotypes and older students were more likely to be dismissed from class (chronotype: b = −0.928, z = −2.285, p = 0.0223; Fig. 2b; age: b = −1.498, z = −2.568, p = 0.0102). Among the students who had been dismissed from class at least once per school year, younger and male students had an increased chance of being dismissed more often (age: b = −0.391, z = −2.685, p = 0.0073; sex: b = 0.831, z = 3.447, p = 0.0006). Chronotype also influenced the frequency and the duration of sick leaves, with late chronotypes being more often and more days sick (sick leave frequency: b = 0.125, t (420) = 2.202, p = 0.0282; Fig. 2c; sick leave duration: b = 0.123, t (420) = 1.975, p = 0.049; Fig. 2d). We did not find a significant effect of sex and age on the number of sick leaves. The complete overview of the effects of chronotype, sex, and age on school attendance is reported in Table 3.

**Table 3:** Influence of chronotype, sex, and age on school attendance variables.

	late arrivals (zero-inflation model)			class dismissals (zero-inflation model)		
explanatory variables	b	z-value	p-value	b	z-value	p-value
chronotype	−0.695	−2.555	0.0106	−0.928	−2.285	0.0223
sex	—	—	—	−0.990	−1.388	0.1651
age	—	—	—	−1.498	−2.568	0.0102
	late arrivals (count model)			class dismissals (count model)		
explanatory variables	b	z-value	p-value	b	z-value	p-value
chronotype	—	—	—	0.192	1.796	0.0725
sex	—	—	—	0.831	3.447	0.0006
age	0.320	4.700	<0.0001	−0.391	−2.685	0.0073
	sick leaves			sick leaves (days)		
explanatory variables	b	t-value	p-value	b	t-value	p-value
chronotype	0.125	2.202	0.0282	0.123	1.975	0.049
sex	−0.224	−1.839	0.0666	−0.217	−1.620	0.106
age	0.064	0.984	0.3258	0.101	1.413	0.158

The model estimates (b coefficients), the test statistic (z-test and t-test), and the p-values associated with each independent variable are reported. For late arrivals and class dismissals both the zero-inflation model and the count model statistics are indicated. If a variable was not included in the final selected model, this is indicated by horizontal dashes (—).

**Figure 3:**
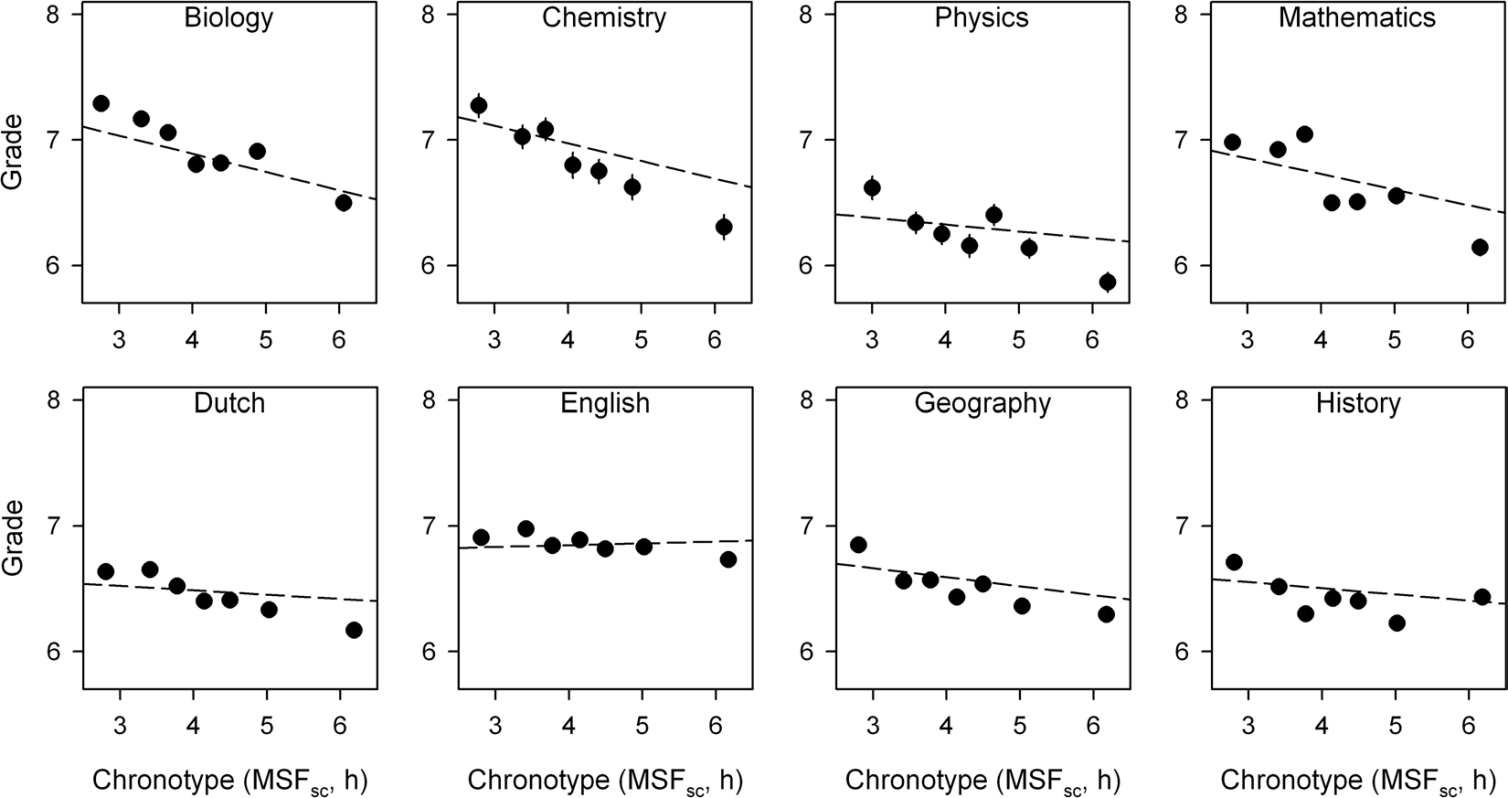
Influence of chronotype (MSF_sc_) on grades by subject. Data points represent mean grades with standard error of the mean (SEM). Since the SEM were very small, error bars are not always visible. Mean grades were calculated for 7 equal-sized groups of students based on chronotype (lower numbers correspond to earlier chronotypes and higher numbers to later chronotypes, respectively). Regression lines representing the association between chronotype and grades (raw data) were calculated with multilevel mixed modelling separately per each subject. The raw data are shown in Supplementary Figure S2. The influence of chronotype on grades was significant only for geography, biology, chemistry, and mathematics.

Finally, we added an interaction effect (chronotype x school subject) to our multilevel model to explore whether the size and significance of effect of chronotype on grades was different between school subjects. We found that the interaction effect was significant (F_7,32920_ = 15.490, p < 0.0001), meaning that the slopes of the regression lines describing the effect of chronotype on grades were significantly different between school subjects. We therefore fitted the multilevel model separately by school subject (Fig. 3).

**Figure 4:**
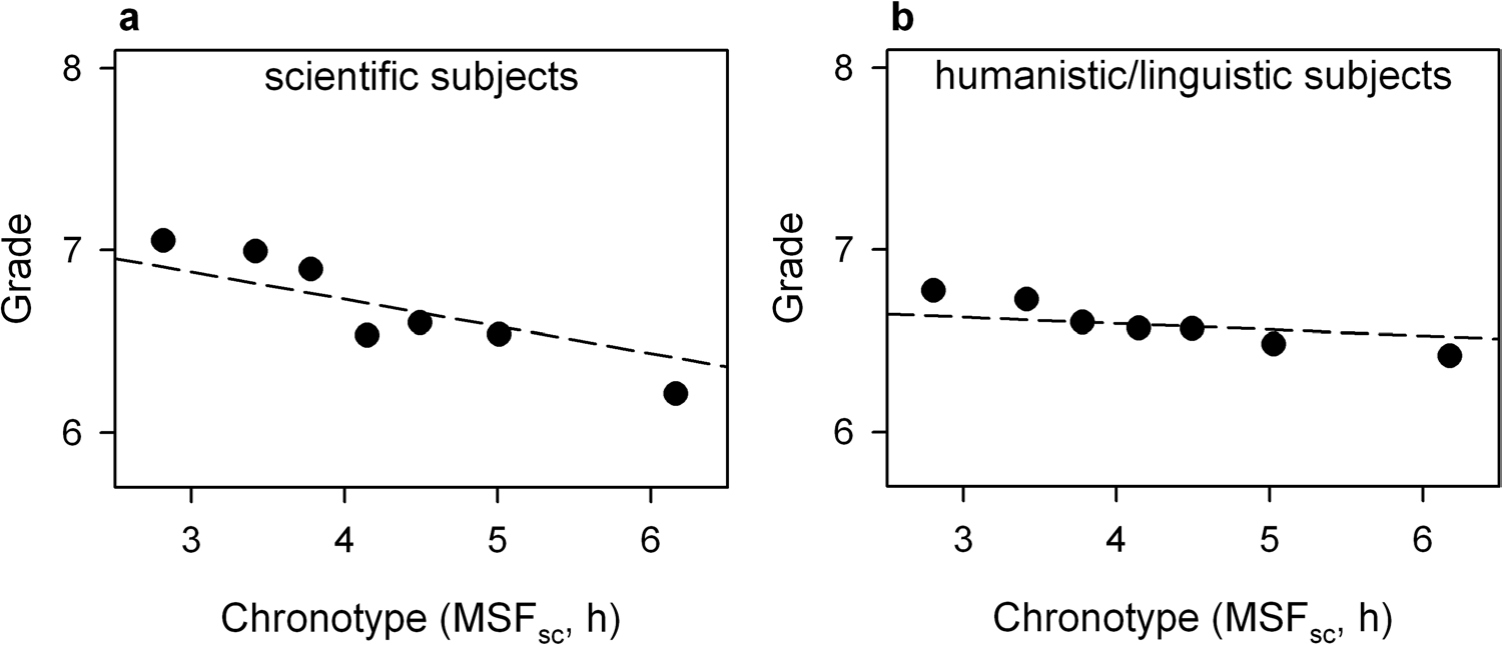
Influence of chronotype (MSF_sc_) on grades by subject area. Data points represent mean grades with standard error of the mean (SEM). Since the SEM were very small, error bars are not always visible. Mean grades were calculated for 7 equal-sized groups of students based on chronotype (lower numbers correspond to earlier chronotypes and higher numbers to later chronotypes, respectively). Regression lines representing the association between chronotype and grades (raw data) were calculated with multilevel mixed modelling separately per each subject. The raw data are shown in Supplementary Figure S3. The influence of chronotype on grades was significantly stronger for scientific subjects (biology, chemistry, physics, and mathematics) (**a**) compared with humanistic/linguistic subjects (Dutch, English, geography, and history) (**b**).

The effect of chronotype on grades was significant for geography (b = −0.071, t (405) = −2.559, p = 0.0108), biology (b = −0.145, t (254) = −3.423, p = 0.0007), chemistry (b = −0.141, t (162) = −2.412, p = 0.0170), and mathematics (b = −0.124, t (405) = −2.543, p = 0.0114), and was not significant for English (b = 0.014, t (405) = 0.315, p = 0.7528), history (b = −0.050, t (405) = −1.316, p = 0.1889), physics (b = −0.058, t (99) = −0.738, p = 0.4621), and Dutch (b = −0.034, t (405) = −1.414, p = 0.1581). In geography, biology, chemistry, and mathematics late chronotypes obtained lower grades compared to early chronotypes. The complete overview of the effects of all variables on grades by subject is reported in Table 4. Based on these results, we divided the school subjects into two groups: scientific (biology, physics, chemistry, and mathematics) and humanistic/linguistic subjects (Dutch, English, history, and geography). The interaction effect between chronotype and subject area (scientific vs. humanistic/linguistic) was significant (Fig. 4; F_1,32932_ = 73.567, p < 0.0001), with the effect of chronotype on grades being significantly stronger for scientific subjects compared to humanistic/linguistic subjects.

**Table 4:** Influence of demographic, sleep-related and school attendance variables on school grades by academic subject.

Explanatory variables	Geography			History			English			Dutch		
	b	t-value	p-value	b	t-value	p-value	b	t-value	p-value	b	t-value	p-value
sex	0.125	2.125	0.0342	0.132	1.651	0.0995	−0.274	−2.857	0.0045	−0.326	−6.446	<0.0001
chronotype	−0.071	−2.559	0.0108	−0.049	−1.316	0.1889	0.014	0.315	0.7528	−0.034	−1.414	0.1581
late arrivals	−0.052	−2.489	0.0132	−0.088	−3.299	0.0011	−0.070	−2.066	0.0395	−0.048	−2.685	0.0075
class dismissals	−0.076	−3.575	0.0004	−0.047	−1.656	0.0985	−0.067	−1.953	0.0515	−0.101	−5.545	<0.0001
sick leaves (days)	−0.014	−2.723	0.0067	−0.022	−3.097	0.0021	−0.012	−1.408	0.1598	−0.014	−3.175	0.0016
	biology			physics			chemistry			mathematics		
explanatory variables	b	t-value	p-value	b	t-value	p-value	b	t-value	p-value	b	t-value	p-value
sex	−0.116	−1.349	0.1787	0.082	0.512	0.6099	0.249	2.164	0.0319	−0.188	−1.823	0.0690
chronotype	−0.145	−3.423	0.0007	−0.058	−0.738	0.4621	−0.141	−2.412	0.0170	−0.124	−2.543	0.0114
late arrivals	−0.062	−1.939	0.0536	0.020	0.510	0.6114	−0.093	−2.074	0.0397	−0.065	−1.846	0.0656
class dismissals	−0.079	−2.152	0.0323	−0.256	−3.313	0.0013	−0.101	−3.018	0.0030	−0.126	−3.435	0.0007
sick leaves (days)	−0.031	−3.910	0.0001	−0.018	−1.525	0.1303	−0.034	−9.296	0.0012	−0.031	−3.290	0.0011

The model estimates (b coefficients), the test statistic (t-test), and the p-values associated with each independent variable are reported.

## Discussion

The aim of the current study was to quantify the impact of chronotype, sleep duration, time of day, and school attendance on school performance. We collected grades and school attendance data in Dutch high-school students over an entire school year. Our results are consistent with other studies, in that chronotype correlates significantly with school grades: late chronotypes obtain, on average, lower grades^10, 21, 34–36^. The strength of the chronotype effect was comparable to the negative effect of absenteeism on grades. A one-hour later chronotype was associated with a decrease in grades with a factor of 0.06 (on a scale from 1 to 10). With a difference of almost 3 hours between the earliest 20% (mean MSF_sc_ = 2:56 h) and the latest 20% (mean MSF_sc_ = 5:53 h) of chronotypes in our sample, the model predicted an overall decrease in grades of 0.18 for late compared to early chronotypes. This represents a difference from the 55^th^ to the 43^rd^ percentile of grades in our data set.

The model selection based on the AIC revealed that sleep duration was one of the weaker predictors among our explanatory variables. Rather chronotype, sex, and school attendance most closely correlated with school grades. Sleep duration was significantly associated with grades only without controlling for other variables such as school attendance and chronotype. In addition, our mediation analysis revealed a significant direct effect of chronotype on grades independent of sleep duration on school days. Comparison between the direct and the indirect (sleep-duration mediated) effect of chronotype showed that the strength of the effect of the former was 5 times larger. Although several reviews have reported an association between short sleep duration and poor school performance^37–40^, our findings suggest that chronotype has a stronger influence on school performance. Since a late chronotype can lead to short sleep duration on school/work days^20^, our work emphasises the need to disentangle these variables in future studies. It is important to mention that we did not collect data about napping behaviour in these students. It is possible that the effect of sleep duration on grades was not evident because some students compensated their daily sleep debt through napping.

Similar to sleep duration, levels of social jetlag were found to be associated with grades only when not controlling for school attendance. Previous studies have shown a relationship between increased levels of social jetlag and lower school/academic performance^41, 42^ and between social jetlag and chronotype^43^. However, within this dataset, chronotype was a stronger predictor of school performance than social jetlag.

In addition, we found that chronotype was significantly associated with school attendance, suggesting that the effect of chronotype on grades could be mediated by school attendance: late chronotypes are more often absent, absenteeism is related to lower school performance, and therefore late chronotypes obtain lower grades. However, the results from the mediation analysis indicated that chronotype had a direct effect on grades independent of school attendance. The direct effect of chronotype on grades was on average 15 times larger than the indirect effect of chronotype on grades mediated by school attendance.

Our analyses showed that the effect of chronotype on grades was only significant for scientific subjects (except for physics) and not for humanistic/linguistic subjects. The absence of an effect in physics might be the result of the smaller number of grades present in the dataset for this subject. This is supported by a similar negative estimate for physics as for the other scientific subjects. In contrast to chronotype, absenteeism was always significantly associated with grades obtained in every school subject. Based on these results, we hypothesise that chronotype and school attendance impact school performance differently. On one hand, absenteeism is likely to impair school performance when a student is learning for all subjects that had been taught while he/she was absent, resulting in lower grades independent of subject. Chronotype, on the other hand, may impact specific cognitive processes that are important for scientific subjects, resulting in lower grades only for these subjects. This hypothesis is supported by previous research showing that both chronotype and time of day have a stronger effect on cognitive performance in tasks requiring fluid intelligence (reasoning, logic, abstract thinking) than on those using crystallised intelligence (general knowledge)^13, 22–25^. Fluid intelligence, in turn, characterises thought processes used for exams in scientific subjects rather than humanistic/linguistic subjects^44, 45^. Studies measuring brain activity with EEG and fMRI also found chronotype and time-of-day variation on tasks involving fluid intelligence^46, 47^.

In contrast to our results, a recent study assessing the effect of diurnal preferences on different school subjects found that eveningness (but not morningness) was negatively associated with grades obtained in both scientific and linguistic subjects^34^. However, both the assessment of chronotype (two-dimensional) and grades (self-reported grades averaged per subject area) used different methods compared to our study, limiting a direct comparison between studies.

For our work, we used an approach based on regression analysis. It is important to note that this, per definition, does not allow investigating causal relationships among variables of interest. In addition, we analysed a specific set of predictors of school performance, and we did not assess other factors, such as academic beliefs and academic motivation, that have been previously found to be associated with school grades^2, 3^. Future studies may therefore include some of these factors. For instance, achievement motivation has been found to mediate the effect of chronotype on grades^11, 48^.

Because of the consistent chronotype-effect on grades described in many studies, future research should focus on investigating the mechanisms underlying this effect so that evidence-based school policies can be implemented. As it stands now, early school starting times are a form of discrimination against late chronotypes^10, 11, 21, 34–36, 49^. Compared to other factors (socio-economic status, academic beliefs, intelligence), adaptation of school schedules to the students’ sleep needs is relatively easy – and the payoff is high. Like these other factors, late chronotype is not a defect or pathology but rather part of the normal variation in the human condition.

Taken together our findings suggest that a change in school schedules would improve school attendance and performance, especially in late chronotypes. There is growing evidence that schools start too early for the circadian clocks of adolescents^10, 17, 19, 50^. More field studies investigating the impact of delayed school starting times are needed, but the first findings are promising in terms of improved students’ sleep, mood, behaviour, school attendance, and performance^51–54^. Our current and previous findings also suggest additional solutions: school schedules could be adapted, favouring examinations later in the day, especially for scientific subjects.

## Methods

### Study protocol

The study was conducted at a Dutch high school in Coevorden (52° 40′N/6° 45′E) between August 2013 and July 2014. The secondary education in the Netherlands is organised in three levels: the VMBO (voorbereidend middelbaar beroepsonderwijs) prepares students for the job market (4 years of education from age 12 to 16); the HAVO (hoger algemeen voortgezet onderwijs) prepares students to study at universities of applied sciences (5 years of education from age 12 to 17); the VWO level (voorbereidend wetenschappelijk onderwijs) prepares students to study at research universities (6 years of education from age 12 to 18). We collected 40,890 school grades in 523 students attending the first three years of secondary school. Between September and November 2013, 426 students filled in the Munich ChronoType Questionnaire (MCTQ^14^). Chronotype was determined (mid-point of sleep on school-free days corrected for sleep debt on school days; MSF_sc_). The MCTQ also allows assessing other sleep-related variables, such as average sleep duration of the week, sleep duration separately on school days and school-free days, and social jetlag. The latter is an approximate quantification of the mismatch between the biological and social clocks^43^.

The school subjects assessed in this study were geography, history, Dutch, English, biology, mathematics, chemistry, and physics. In the Dutch secondary school system, grades range from 1 (lowest) to 10 (highest), with 6 considered to be the threshold to pass an examination^32^. Grades were collected during 4 periods (Fall: August - October; Winter: November - January; Spring: February - April; Summer: May - July). Students from a total of 20 classes participated in the study. These spanned two levels: the HAVO and the VWO. 12 of the 20 classes belonged to the HAVO, and 8 classes were drawn from the VWO. An overview of all classes by level and by year of education is reported in the Supplementary Table S1. This hierarchy in school levels was mirrored in our analysis using a multilevel approach with students nested within classes, and classes nested within levels of education. Late arrivals, dismissals from class (when a student due to misbehaviour was sent out of class by the teacher), frequency of sick leaves and duration of each sick leave in days were extracted from the school’s registration system.

### Statistical analysis

Statistical analyses were done using R software version 3.3.0^55^. A multilevel approach was used to explain the effects of the independent variables on school grades (dependent variable). The independent variables assessed were: demographic variables (sex and age), school attendance variables (late arrivals during the first hour, dismissals from class at any time of day, sick leaves, and sick leave duration), and sleep-related variables (chronotype; MSF_sc_), social jetlag, and sleep duration on school days. We built nine multilevel models, each with a different subset of explanatory variables. Student ID was included as a random factor nested within class and within level of education (HAVO and VWO). School subject (geography, history, Dutch, English, biology, mathematics, chemistry, and physics) and time of year/season when the grades were collected (Fall: August - October; Winter: November - January; Spring: February - April; Summer: May - July) were entered in all models, and analysed as covariates. Model selection based on the Akaike Information Criterion (AIC^56^) was performed to select the best combination (fit) of independent variables explaining the variation in school grades. The most parsimonious model is defined as the model with the lowest AIC value. We used the guidelines of Kass and Raftery to compare models^57^. The estimates of the model are indicated in the results as “b” coefficients. To compare the strength of the effects of the different predictors the coefficients were standardised and are indicated as “β” coefficients.

To further explore the relationship between chronotype, school attendance, sleep duration, and school performance, we ran 4 separate mediation analyses with chronotype as predictor, and late arrivals, dismissals from class, sick leaves, and sleep duration on school days as mediators. Average grades over the entire school year were used as the dependent variable. Sex and class were analysed in these models as covariates. General linear models with Poisson regression were used to analyse the relationship between chronotype and school attendance variables. Linear models were used to analyse the relationship between chronotype and sleep duration, and between chronotype and average grades. Analyses were done using the R package for causal mediation analysis^58^.

The relationship between chronotype and school attendance variables was modelled taking into account the distribution of data for the specific variables of interests (late arrivals, dismissals from class, sick leaves and sick leave duration). Zero inflation models (to account for the high frequency of zero values) with negative binomial distribution (to account for over-dispersion of the data) were chosen to model the effect of chronotype on late arrivals and dismissals from class. Zero inflation models combine two processes: the first model predicts whether an event has occurred or not and is governed by a binary distribution; the second model predicts how many times an event is likely to occur and is governed by a Poisson distribution (count data). General linear models with quasi-Poisson regression were used to test the effect of chronotype on frequency and duration of sick leaves. Sex and age were added to the models as covariates. Model selection based on AIC was again applied.

### Ethical approval and informed consent

The study was conducted according to the principles of the Medical Research Involving Human Subjects Act (WMO, 2012), and the Declaration of Helsinki (64^th^ WMA General Assembly, Fortaleza, Brazil, October 2013). The Medical Ethical Committee of the University Medical Centre of Groningen (NL) and the head of the school approved the study. Written informed consent was obtained from the head of the school.

### Data availability

The datasets generated and analysed during the current study are available at the public data depository Dataverse (http://hdl.handle.net/10411/A7RTLA).

## Electronic supplementary material

Supplementary Information
