# Supplementary material for: Lower school performance in late chronotypes: underlying factors and mechanisms
Source: Sci Rep. 2017 Jun 29;7:4385. doi: 10.1038/s41598-017-04076-y (PMC5491513; doi:10.1038/s41598-017-04076-y)
Supplement: Supplementary file 1 — Supplementary Information [file 41598_2017_4076_MOESM1_ESM.pdf]

Lower school performance in late chronotypes: underlying factors and mechanisms

Giulia Zerbini, Vincent van der Vinne, Lana Otto, Thomas Kantermann, Wim Krijnen, Till Roenneberg, and Martha Merrow

Supplementary Information

Supplementary Table S1. Overview of the classes by level and by school year of education.

	HAVO	VWO
1st year	1h1, 1h2, 1h3, 1h4	1a1, 1a2, 1g1
2nd year	2h1, 2h2, 2h3, 2h4	2a1, 2a2, 2g2
3rd year	3h1, 3h2, 3h3, 3h4	3a1, 3ag1

Supplementary Table S2. Description of the 9 models used to explore the influence of demographic, sleep-related, and school attendance variables on school grades.

Model	Explanatory variables	K	AICc	Delta AICc	AICcWt	ER	LER
1	sex, chronotype, late arrivals, dismissals from class, sick leaves (d)	20	118909.8	0.86	3.01 e-1	1.54	0.19
2	sex, social jetlag, late arrivals, dismissals from class, sick leaves (d)	20	118911.8	2.86	1.11 e-1	4.17	0.62
3	sex, sleep duration on school days, late arrivals, dismissals from class, sick leaves (d)	20	122818.4	3909.36	0	n.a.	n.a.
**4**	**sex, age, chronotype, late arrivals, dismissals from class, sick leaves (d)**	**21**	**118909**	**0**	**4.61 e-1**	**1**	**0**
5	sex, age, social jetlag, late arrivals, dismissals from class, sick leaves (d)	21	118911.6	2.57	1.27 e-1	3.62	0.56
6	sex, age, sleep duration on school days, late arrivals, dismissals from class, sick leaves (d)	21	122816.9	3907.89	0	n.a.	n.a.
7	sex, age, chronotype	18	118959.3	50.29	5.50 e-12	83,895,618,181.82	10.92
8	sex, age, social jetlag	18	118964.2	55.25	5.00 e-13	922,851,800,000.00	11.96
9	sex, age, sleep duration on school days	18	122880.9	3971.90	0	n.a.	n.a.

The number of estimated parameters (K) for each model is reported. The most parsimonious model for the given data was model 4 (lowest AICc score = 118909). The differences in AICc scores (delta AICc) are calculated for each model relative to model 4. The weight of each model (AICcWt) indicates the weight of evidence for that model to be the best fit for the given data. Model 4 received 46% of the total weight of the models considered. The evidence ratio (ER) is the ratio between the weight of the best model and the weight of each single model. The log evidence ratio (LER) can be used to compare the models. We used Kass and Raftery[57] guidelines to compare the models: LERs greater than 0, 0.5, 1, and 2 indicate respectively "minimal", "substantial", "strong", and "decisive" evidence for model 4 to be the most parsimonious relative to the other models. There was minimal evidence for model 4 to be a better fit of the data compared to model 1. Model 4 had the same set of predictors as model 1, with age as additional predictor. Since age was not significantly related to grades ($b = 0.084$, $t(406) = 1.802$, $p = 0.0723$), we described the effect on grades of the set of predictors in model 1. There was substantial evidence for model 4 to be a better fit of the data compared to model 2 and 5 that had social jetlag among the set of predictors. Social jetlag was not significantly related to grades (model 2: $b = -0.051$, $t(407) = -1.835$, $p = 0.0672$; model 5: $b = -0.055$, $t(406) = -1.960$, $p = 0.0507$). There was decisive evidence for both model 4 and 1 (models with chronotype among the predictors) to be a better fit of the data compared to model 3 and 6 that had sleep duration among the predictors, and compared to models 7, 8, and 9 that did not have the school attendance variables among the predictors.

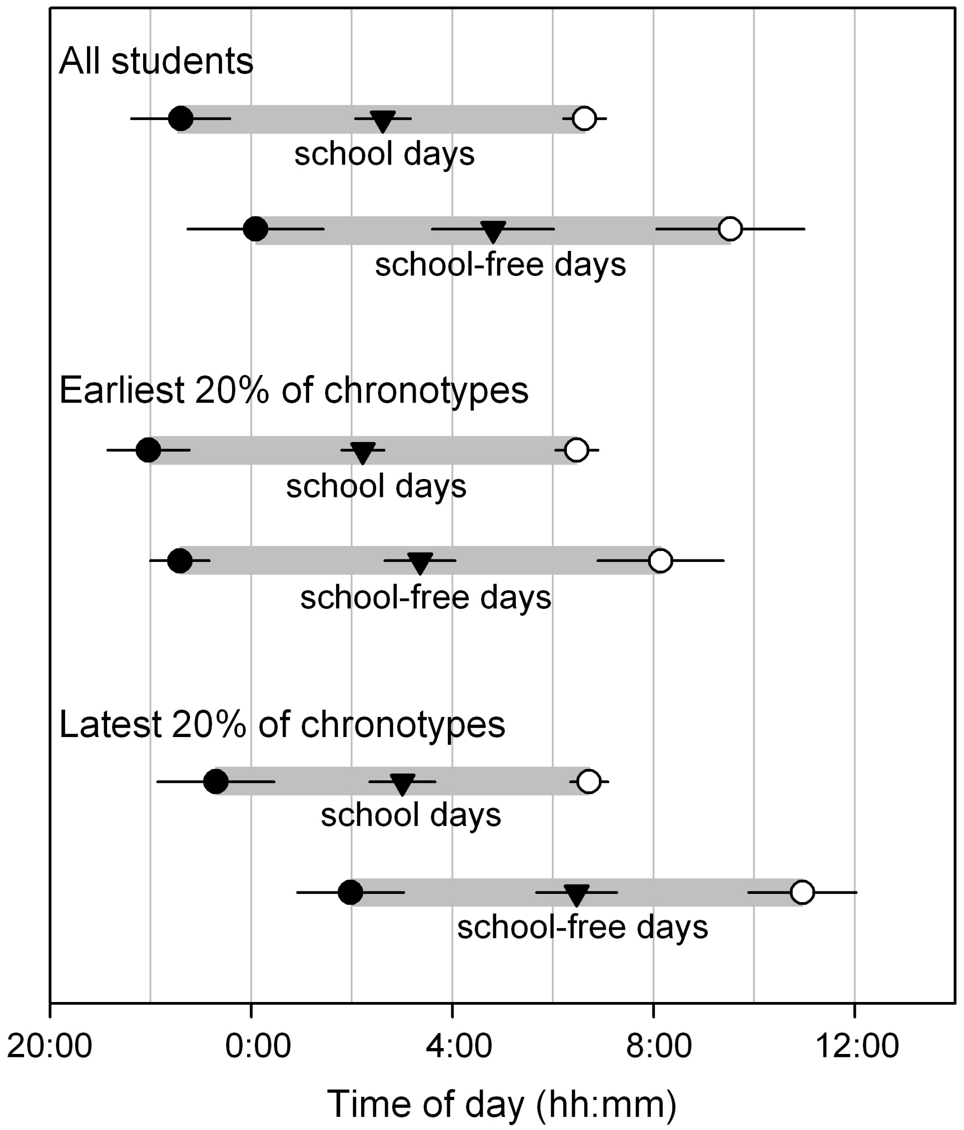

Supplementary Figure S1. Sleep characteristics of all students and of the 20% earliest and 20% latest chronotypes (data from the MCTQ).
Data points represent averages of sleep onset (filled circle), midpoint of sleep (filled triangle), and sleep end (open circle) on school days and on school-free days. Error bars represent standard deviations.

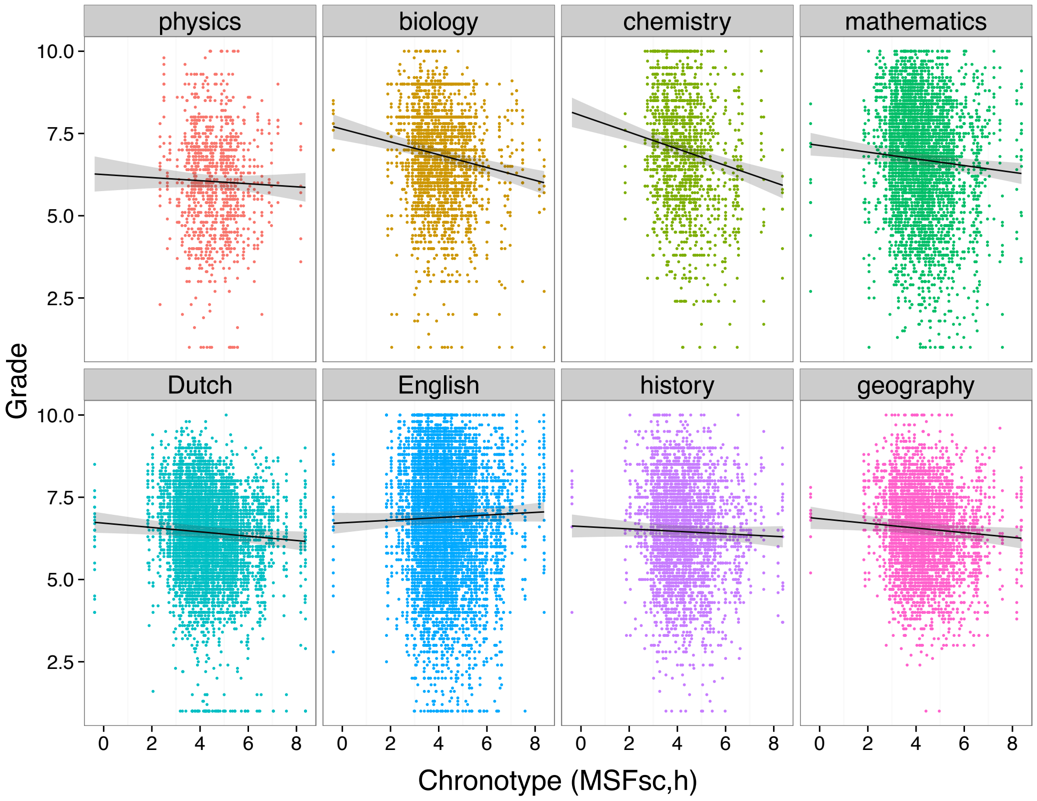

Supplementary Figure S2. Influence of chronotype (MSF_{sc}) on grades by subject.
Data points represent individual grades. The fits of the model with confidence interval (grey area) are plotted. The influence of chronotype on grades was significant only for geography, biology, chemistry, and mathematics.

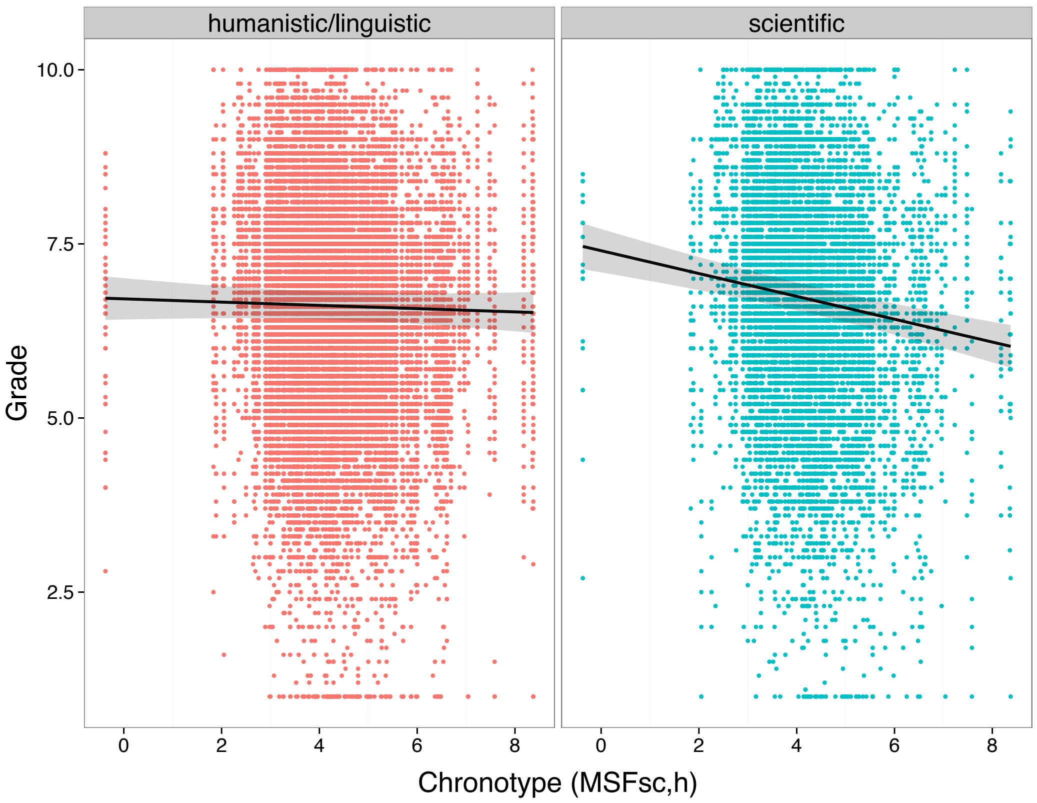

Supplementary Figure S3. Influence of chronotype (MSF_{sc}) on grades by subject area.
Data points represent individual grades. The fits of the model with confidence interval (grey area) are plotted. The influence of chronotype on grades was significantly stronger for scientific subjects compared with humanistic/linguistic subjects.
